# Oxidative stress-induced EGR1 upregulation promotes NR4A3-mediated nucleus pulposus cells apoptosis in intervertebral disc degeneration

**DOI:** 10.18632/aging.205920

**Published:** 2024-06-28

**Authors:** Si-Kuan Zheng, Xiao-Kun Zhao, Hui Wu, Ding-Wen He, Long Xiong, Xi-Gao Cheng

**Affiliations:** 1 Department of Orthopedics, The Second Affiliated Hospital, Jiangxi Medical College, Nanchang University, Nanchang, Jiangxi 330006, China; 2 Institute of Orthopedics of Jiangxi Province, Nanchang, Jiangxi 330006, China; 3 Institute of Minimally Invasive Orthopedics, Nanchang University, Nanchang, Jiangxi 330006, China

**Keywords:** intervertebral disc degeneration, EGR1, NR4A3, nucleus pulposus, apoptosis

## Abstract

This study aimed to reveal the specific role of early growth response protein 1 (EGR1) and nuclear receptor 4A3 (NR4A3) in nucleus pulposus cells (NPCs) and the related molecular mechanism and to identify a new strategy for treating intervertebral disc degeneration (IVDD). Bioinformatics analysis was used to explore and predict IVDD-related differentially expressed genes, and chromatin immunoprecipitation sequencing (ChIP-seq) revealed NR4A3 as the EGR1 target gene. An *in vitro* NPC model induced by tributyl hydrogen peroxide (TBHP) and a rat model induced by fibrous ring acupuncture were established. Western blotting, quantitative real-time polymerase chain reaction (qRT-PCR), immunohistochemical staining, immunofluorescence staining, and flow cytometry were used to detect the effects of EGR1 and NR4A3 knockdown and overexpression on NPC apoptosis and the expression of extracellular matrix (ECM) anabolism-related proteins. Interactions between EGR1 and NR4A3 were analyzed via ChIP-qPCR and dual luciferase assays. EGR1 and NR4A3 expression levels were significantly higher in severely degenerated discs (SDD) than in mildly degenerated discs (MDD), indicating that these genes are important risk factors in IVDD progression. ChIP-seq and RNA-seq revealed NR4A3 as a direct downstream target of EGR1, and this finding was verified by ChIP-qPCR and dual luciferase reporter experiments. Remarkably, the rescue experiments showed that EGR1 promotes TBHP-induced NPC apoptosis and impairs ECM anabolism, dependent on elevated NR4A3 expression. In summary, the EGR1-NR4A3 axis mediates the progression of NPC apoptosis and ECM impairment and is a potential therapeutic target in IVDD.

## INTRODUCTION

Intervertebral disc degeneration (IVDD) is a significant cause of lower back pain (LBP) that greatly impacts patients’ daily lives [1, 2]. The intervertebral disc (IVD) is a fibrous cartilage tissue located between adjacent vertebral bodies and comprises the nucleus pulposus (NP), annulus fibrosus (AF), and cartilage endplate. Previous studies have shown that nucleus pulposus cells (NPCs) surrounded by extracellular matrix (ECM) play a crucial role in regulating IVDD [3]. Patients with IVDD accompanied by nerve or spinal cord compression symptoms often require surgical treatments, such as minimally invasive spinal surgery, intervertebral fusion surgery, or total disc replacement surgery. However, these surgical interventions directly damage the original IVD structure and often cause complications, such as recurrent neurological symptoms and adjacent segment disc degeneration [4, 5]. The main characteristics of IVDD are the reduction or dysfunction of NPCs and the loss of ECM [6, 7]. Therefore, studying the pathological mechanism underlying the reduction in NPCs in IVDD and exploring potential therapeutic targets has important clinical and social significance.

Located within the human chromosome 5q23–31 region, early growth response protein 1 (EGR1) represents a crucial member of the early growth response protein family [8]. As a pivotal transcription factor, EGR1 encompasses an activation domain, an inhibitory region, and trio Cys2 His2-type zinc finger domains. EGR1 specifically recognizes and binds target genes and regulates their transcription [8]. EGR1 is widely expressed in many cell types and participates in important physiological processes, such as cell proliferation, differentiation, invasion, and apoptosis [9–11]. When cells are stimulated by factors such as oxidative stress, growth factors, or inflammatory cytokines, EGR1 is rapidly and transiently induced to either promote or inhibit the transcription of downstream genes [12]. Previous studies have shown that EGR1 is upregulated in osteoarthritis and accelerates chondrocyte hypertrophy-induced arthritis [13]. However, the function and specific mechanism of EGR1 in intervertebral disc NPCs remain unclear.

Our research identified NR4A3 as a direct downstream target of EGR1. Nuclear receptor subfamily 4 group A member 3 (NR4A3) is an important regulator of cellular function [14, 15]. Although NR4A3 is expressed in various cell types, there is insufficient research on the function of this molecule. Recent studies have shown that NR4A3 is upregulated in cultured Ins-1 cells and human pancreatic islets treated with IL-1β and TNFα. NR4A3 knockdown can damage cytokine-mediated cell apoptosis, and NR4A3 overexpression can induce cell apoptosis [16]. These data indicate that NR4A3 has a pro-apoptotic effect. One study demonstrated that the NR4A family affects ECM protein synthesis by regulating NF-κB signal transduction in bone marrow cells [17]. Another suggested that NR4A3 is highly expressed in osteoarthritis and can promote inflammation and the expression of cartilage matrix degradation genes [18]. However, the biological function of NR4A3 has not been investigated in IVDD research.

Hence, the primary objective of this study was to explore the roles and molecular mechanisms of EGR1 and NR4A3 in the progression of IVDD.

## MATERIALS AND METHODS

### Patient samples

Ten intervertebral disc NP tissue specimens were collected from patients who underwent surgery at the Second Affiliated Hospital of Nanchang University between September 2021 and June 2023. The patients’ preoperative spinal magnetic resonance imaging (MRI) data were used to grade discs; those determined to be level I or II according to the Pfirrmann classification were categorized into the control group (MDD) and were obtained from young patients with acute disc herniation or newly developed traumatic spinal fractures; discs graded as level III, IV, or V were categorized into the degenerative group (SDD) and were taken from patients diagnosed with degenerative disc disease. Follow-up visits were conducted for each patient, and clinical data were comprehensively collected, encompassing details such as patient name, age, sex, diagnosis, MRI results, body mass index (BMI), and other pertinent information (Supplementary Table 1). A trio of observers independently scrutinized the extent of IVDD using the Pfirmmann classification system. This study protocol was approved by the Ethics Committee of the Second Affiliated Hospital of Nanchang University, and written informed consent was obtained from each patient.

### NPC culture and induction

The immortalized human NP cell line was procured from ScienCell Research Laboratories, Inc. NPC medium (ScienCell Research Laboratories, Carlsbad, CA, USA) supplemented with 10% fetal bovine serum and 1% penicillin/streptomycin was used to culture the NP cells. The optimal culture conditions were 37°C within a humidified chamber with 5% atmospheric CO_2_. An *in vitro* NPC oxidative stress degeneration model was induced by treating the cells with 50 μM tert-butyl hydroperoxide (TBHP) for 24 hours [19, 20].

### Quantitative real-time polymerase chain reaction (qRT-PCR)

Total RNA was isolated from NPCs using TRIzol reagent (TRANS, China) according to the manufacturer’s instructions. Subsequently, 500 ng of total RNA was reverse transcribed into cDNA with an RT reagent kit (Takara Bio Co., Ltd., China). qRT-PCR analysis was performed using the TB Green Premix qPCR Kit (Takara Bio Co., Ltd., China) on the Bio-Rad CFX Connect System (Bio-Rad, CA, USA). The results were calculated using relative expression levels and the 2^−ΔΔCt^ method. The primers used for amplification are as follows: Homo EGR1 (Forward 5′-CTAGTGAGCATGACCAACCCAC-3′, Reverse 5′-CGCTGAGTAAATGGGACTGCTGT-3′), Homo NR4A3 (Forward 5′-CAACAGGAACCTTCTCAGCCCTC-3′, Reverse 5′-ATGGAGGCTGTCAGGAGGTTGTA-3′), Homo GAPDH (Forward 5′-TGACTTCAACAGCGACACCCA-3′, Reverse 5′-CACCCTGTTGCTGTAGCCAA-3′), Rattus EGR1 (Forward 5′-CCATCACCTATACTGGCCGCTTC-3′, Reverse 5′-AGGTGGGTGCAGCTGAGTAAATG-3′), Rattus GAPDH (Forward 5′-GCCCAGAACATCATCCCTGCAT-3′, Reverse 5′-GCCTGCTTCACCACCTTCTTGA-3′).

### *In vitro* siRNA transfection

NPCs were plated in six-well plates and transfected with scrambled EGR1 siRNA or NR4A3 siRNA using Lipo3000 Transfection Reagent (Beijing Ju-Mei Biotechnology Co., Ltd., China) according to the manufacturer’s instructions. After 48 hours, cellular lysates were isolated to determine the expression of target genes. The siRNAs were purchased from GenePharma Co., Ltd. (China). The target sequences of EGR1 and NR4A3 are as follows: Homo si-EGR1-1 (GGACAAGAAAGCAACAAA), Homo si-EGR1-2 (GGCAUACCAAGAUCCACUU), Homo si-EGR1-3 (GGACCUGAAGGCCCUCAAU), Homo siNR4A3-1 (GCACUCCAUGUACUUCAAGC), Homo siNR4A3-2 (GCUUGAAGUACAUGG AGGUGC), Homo siNR4A3-3 (CGAUGUCAGUACUGUCGAUUU), Rattus si-EGR1 (GGACAAGAAAGCAGACAAA).

### Western blotting

NPCs were lysed in an ice-cold lysis buffer (Solarbio Co., Ltd., China) and centrifuged for 15 min at 4°C, after which the supernatant was collected as the whole-cell extract. The protein content was measured using a BCA assay kit (Bioshar Co., Ltd., China), and 10 μg of total protein from each sample was separated on a 10% sodium dodecyl sulfate–polyacrylamide gel and then transferred onto a polyvinylidene fluoride membrane (Millipore, Germany). The membrane was then blocked with a solution of 5% skim milk in phosphate-buffered saline (PBS) for 1 h at room temperature before being incubated with primary antibodies against EGR1 (1:1000, Proteintech, China), NR4A3 (1:1000, Proteintech, China), GAPDH (1:5000, Proteintech, China), Bax (1:2000, HUABIO, China), Bcl-2 (1:2000, Proteintech, China), Caspase-3 (1:2000, Proteintech, China), Collagen II (1:2000, HUABIO, China), and Aggrecan (1:2000, Affinity, China) at 4°C overnight. The membranes were then washed three times for 10 min each with a solution of 0.05% Tween-20 in PBS. The membranes were subsequently incubated with secondary antibodies for 1 h at room temperature and probed using an enhanced chemiluminescence (ECL) detection reagent. The protein bands were visualized using an ECL detection system (Tanon Science and Technology Co., Ltd.) and analyzed using ImageJ Software.

### Detection of intracellular reactive oxygen species (ROS)

NPCs were cultured in T25 flasks. The experimental group cells were pretreated with TBHP (50 μM, 24 h). The culture medium was removed after treatment, and the cells were washed once with PBS. According to the kit instructions (Bestbio Co., Ltd., China), a 37°C prewarmed DCFDA (008 probe) working solution was added, and the mixture was incubated in the dark for 20–60 minutes. After incubation, the cells were washed with serum-free medium, and PBS was added for imaging under a fluorescence microscope.

### Immunofluorescence staining analysis

NPCs (5 × 10^4^ cells per well) were cultured on a confocal plate and incubated at 37°C. After 24 h, the cells were fixed with 4% paraformaldehyde (400 μl/well) for 15 min, incubated in 0.5% Triton X-100/PBS for 15 min, blocked with 10% goat serum for 30 min at room temperature, and incubated with an anti-EGR1 rabbit polyclonal antibody (1:500, Proteintech, China) overnight at 4°C. After removal of the primary antibody by washing with PBS for 30 min, the cells were incubated with Alexa 488-conjugated secondary antibodies (1:500, Abcam, USA) for 1 h in the dark at room temperature. After washing with PBS for 30 min, the sections were stained with a DAPI solution for 5 min to detect the nucleus. Images of the sections were obtained with a fluorescence microscope (Olympus, Japan).

### Flow cytometry

NPCs were seeded into 6-well plates and subjected to various pretreatments, such as treatment with TBHP or transfection, depending on the experimental requirements. Following pretreatment, the cells were digested with trypsin, and approximately 5 × 10^5^ cells were collected and resuspended. NPCs were then centrifuged and washed with PBS to remove the supernatant per the kit manufacturer’s directions (Elabscience, China). The cell pellet was resuspended in 100 μL of diluted Annexin V Binding Buffer. Annexin V-FITC (2.5 μL) and propidium iodide (PI, 2.5 μL, 50 μg/mL) were added to this suspension. The mixture was incubated in the dark at room temperature for 20 minutes for staining. After incubation, 400 μL of diluted Annexin V binding buffer was added, and the sample was thoroughly mixed. Dual-parameter analysis was conducted using a flow cytometer (Beckman Coulter, Inc., DxFLEX, USA), with the FITC channel set to detect Annexin V-FITC and the ECD channel set to detect PI. Gating strategies for cells double-stained with Annexin V-FITC and PI were used to distinguish among live cells, early apoptotic cells, late apoptotic cells, and necrotic cells. Annexin V^−^/PI^−^ (double-negative) indicated live cells; Annexin V^+^/PI^−^ (positive/negative) identified early apoptotic cells; Annexin V^+^/PI^+^ (double positive) marked late apoptotic or dead cells; and PI^+^ (single positive) was used to identify necrotic cells. Following data acquisition, flow cytometry analysis software (version 10, FlowJo, LLC) was used to evaluate the proportion of apoptotic cells.

### RNA-seq

Human NPCs were divided into the EGR1 overexpression and control groups. Total RNA was extracted from NPCs, and RNA integrity was evaluated using an RNA Nano 6000 Assay Kit. Then, the mRNA was purified from total RNA using poly-T and amplified using PCR technology to establish the RNA database. Finally, the PCR products were purified (AMPure XP system), and library quality was assessed on an Agilent Bioanalyzer 2100 system.

### ChIP-seq

Human NPCs were divided into the EGR1 overexpression and control groups. Approximately 5 × 10^7^ NPCs were treated with 1% formaldehyde to initiate cross-linking, followed by sonication in lysis buffer. Immunoprecipitation was conducted with monoclonal antibodies against EGR1 (Cell Signaling, Inc., USA), followed by ChIP-seq. After the raw sequencing data (raw data) were obtained, the ChIP-seq data were filtered, and the high-quality sequencing data (clean data) were compared with the human genome (hg38). DeepTools software was used to visualize the coverage range of ChIP-seq data, peak annotations, average profiles, and heatmaps of peak-bound TSS regions. Significant enrichment of peaks was detected in the NR4A3 (ENST00000033847) promoter region using the R package GVIZ (https://bioconductor.org/packages/release/bioc/html/Gviz.html), and EGR1 effective binding sites were identified on Jaspar.

### ChIP-qPCR

Human NPCs were collected and cross-linked by adding 1% formaldehyde for 15 min at room temperature. Then, the cells were incubated with glycine at room temperature for 5 minutes to terminate cross-linking and washed twice with PBS. Immunoprecipitation was conducted with 10 μM anti-EGR1 antibody (Cell Signaling, Inc., Rabbit. no. 4153s, USA) and 2 μg of IgG antibody (Cell Signaling Inc., cat. no. 2729s, USA) according to the manufacturer’s instructions (Millipore, MAGNA0017, Germany), followed by qRT-PCR. The primer information is shown in Supplementary Table 2.

### Luciferase assay

The wild-type and mutant plasmids containing the NR4A3 promoter were constructed by Focus Bioscience Co., Ltd., in Nanchang, China. To analyze NR4A3 promoter activity, NPCs in 24-well plates were transfected with DNA containing the NR4A3 promoter and EGR1 using Lipofectamine TM 2000 (Invitrogen, Inc., USA). After 36 h of transfection, the activity of firefly and Renilla luciferases in NPCs was detected using the Dual Luciferase Reporter Gene Assay Kit (Promega, Inc., USA).

### IVDD rat model

An IVDD rat model was established in Sprague-Dawley rats through fibrous ring (AF) acupuncture. Briefly, the rats were anesthetized with 1% sodium pentobarbital (100 mg/kg). After anesthesia, the tail was disinfected with iodophor, and the intervertebral disc was punctured at the level of Co6–7 at a depth of 5 mm using a 21G puncture needle. The needle was inserted into the center of the nucleus pulposus through a fibrous ring and rotated 360° for 30 seconds. EGR1 siRNA (si-EGR1 (2′ O-methyl (OMe) + 5′cholesterol (chol)) and control siRNA (si-Control (2′ OMe + 5′chol-modified)) (RiboBio Co., Ltd., China) were injected into Co6–7 IVDs (5 nmol; 10 μL) at 1, 7, and 14 days after surgery. Four weeks after surgery, a X-ray examination was performed on the rats to confirm the successful establishment of the model. All procedures were approved by the Animal Ethics Committee of Nanchang University.

### X-ray analysis

Preoperatively and 4 weeks postoperatively, the rats underwent X-ray analysis to determine changes in intervertebral height. In brief, the rats were placed in a prone position with their tail straight and the light perpendicular to the tail. The intervertebral disc height index (DHI) was calculated using the method described by Chen et al. [21]. The DHI measured four weeks postoperatively was compared with that measured preoperatively and is expressed as %DHI = (DHI at 4 weeks postoperatively/preoperative DHI ×100%). All images were measured by three independent observers who were unaware of the experimental design.

### Histological analysis

The NP tissues were fixed in 4% paraformaldehyde for 24 hours, decalcified, embedded in paraffin, and sectioned. For immunohistochemical (IHC) analysis, the sections were incubated with an EGR1 antibody (1:200, Proteintech), an NR4A3 antibody (1:200, Proteintech), a collagen II antibody (1:200, HUABIO), and an aggrecan antibody (1:200, Affinity) according to the manufacturer’s instructions. Complete rat disc sections were stained with hematoxylin–eosin (H&E) and saffron O-fast green (SO), and images of the sections were obtained under a microscope (Olympus, Japan). The degree of disc degeneration was assessed according to the historical grading of the disc degeneration [22]. The scores were obtained by three independent observers.

### Statistical analysis

All experiments were performed in triplicate. The data are presented as the mean ± standard deviation (SD). Graphics and statistical analyses were conducted with GraphPad Prism 9.0 software. Student’s *t*-test was used to compare two groups, and one-way ANOVA with Tukey’s post hoc test was used to compare three or more groups. A *p*-value < 0.05 was considered to indicate significance.

## RESULTS

### IVDD-associated genes discovered by microarray

**Figure 1:**
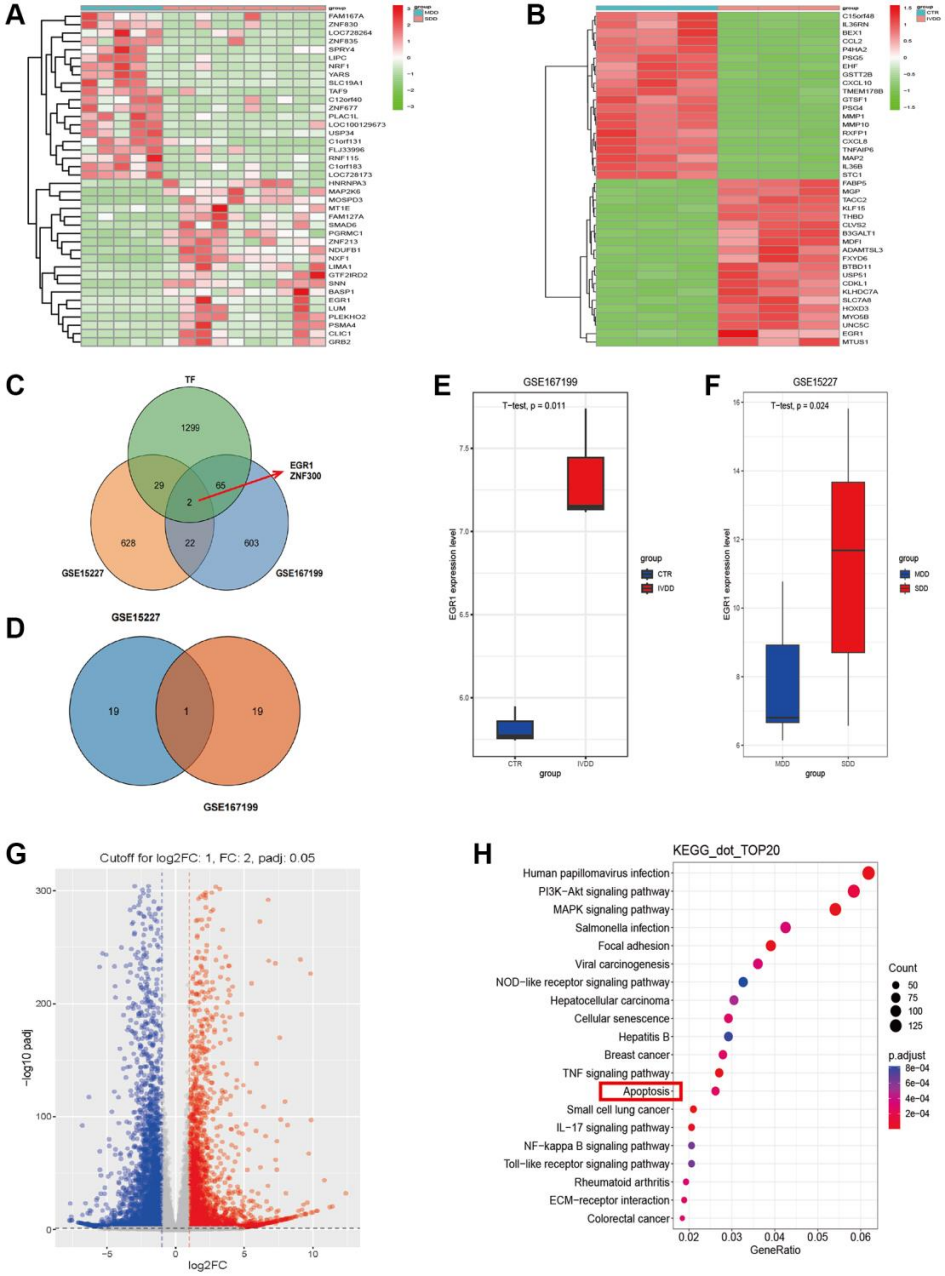
**Abnormally expressed genes associated with IVDD identified using the GSE167199 and GSE15227 datasets.** (**A**, **B**) Heatmap of all differentially expressed genes in degenerative intervertebral disc and nondegenerative intervertebral disc tissues from the GSE167199 and GSE15227 datasets. (**C**) Venn diagram showing the two selected transcription factors. (**D**) early growth response protein 1 (EGR1) was identified as a key gene. (**E**, **F**) EGR1 expression was analyzed by using the GSE167199 and GSE15227 datasets. (**G**) A volcano plot was created using RNA-seq data to show differentially expressed genes. (**H**) Bubble chart showing the KEGG pathway analysis of the RNA-seq data.

To examine the biological role of differentially expressed mRNAs (DEMs) in IVDD, we first analyzed the microarray profiles (GSE15227 and GSE167199) of 13 degenerative NP tissue samples and 8 nondegenerative NP tissue samples (Figure 1A, 1B). We identified 628 upregulated DEMs in the GSE15227 dataset and 603 upregulated DEMs in the GSE167199 dataset. By intersecting the two sets of upregulated differentially expressed genes that we identified with the known set of transcription factors (TFs), we obtained two candidate genes: EGR1 and ZNF300 (Figure 1C). We sorted the upregulated transcription factors with differential expression in the two datasets according to their log FC values from high to low, selected the top 20 TFs from each dataset, and calculated their intersection. Finally, we identified EGR1 as the key TF associated with IVDD (Figure 1D). We further validated the stable upregulation of EGR1 through two datasets (Figure 1E, 1F). Moreover, we performed RNA-seq on NPCs overexpressing EGR1, revealing that EGR1 is involved in various biological processes, including the apoptotic pathway (Figure 1G, 1H). Therefore, we speculate that EGR1 potentially influences IVDD progression.

### EGR1 expression is increased in human degenerative intervertebral discs and TBHP-induced NPCs

**Figure 2:**
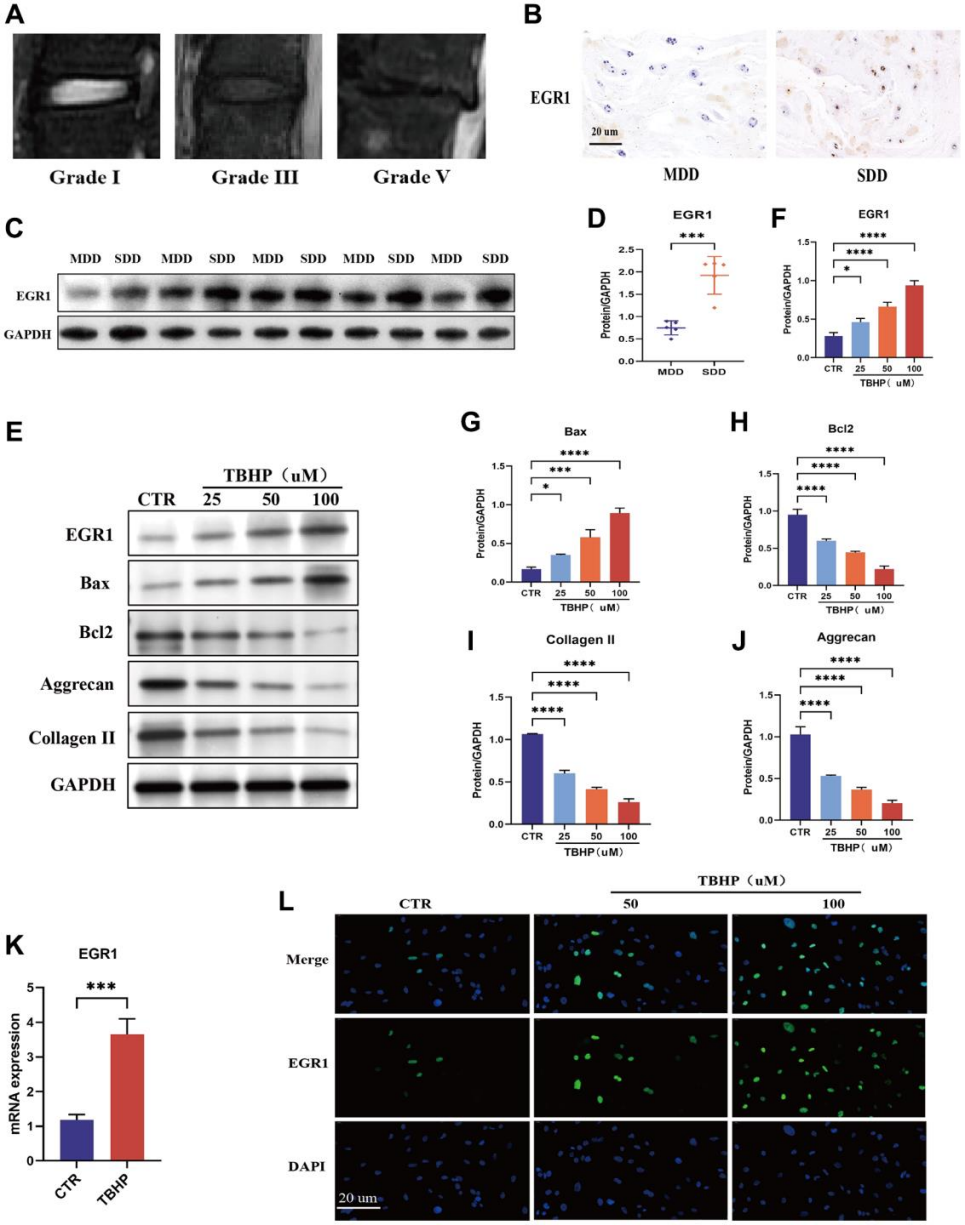
**EGR1 expression is upregulated in human degenerative intervertebral disc tissue and in TBHP-treated NPCs.** (**A**) Representative magnetic resonance images showing different grades of degeneration in human tissue: mild (grades I and II) and severe (grades III–V). (**B**) EGR1 protein expression levels were examined in severely degenerated discs (SDD) and mildly degenerated discs (MDD) by immunohistochemical (IHC) staining (magnification: ×400). (**C**, **D**) The EGR1 expression level in human nucleus pulposus (NP) tissue was analyzed by western blotting. ImageJ Software was used for quantitative analysis, and the data were normalized to GAPDH. (**E**–**J**) Human nucleus pulposus cells (NPCs) were treated with different concentrations (0, 25, 50, or 100 μM) of TBHP for 24 h, after which the proteins were extracted and the expression levels of EGR1, Bax, Bcl-2, aggrecan, and collagen II were determined by western blotting. (**K**) Human NPCs were treated with 50 μM tributyl hydrogen peroxide (TBHP) for 24 h, and EGR1 mRNA expression was examined by qRT-PCR. (**L**) EGR1 protein expression was examined in TBHP-treated NPCs by immunofluorescence staining. ^*^p < 0.05, ^***^p < 0.001, ^****^p < 0.0001.

Representative magnetic resonance images of human intervertebral disc with different degrees of degeneration were obtained (Figure 2A). To investigate the role of EGR1 in IVDD, we first measured its expression in mildly (grades I and II) and severely (grades III, IV, and V) degenerated human intervertebral discs. As expected, immunohistochemistry (IHC) showed that the expression of EGR1 was significantly greater in severely degenerated discs (SDD) than in mildly degenerated discs (MDD) (Figure 2B). Western blot analysis further confirmed that EGR1 protein levels were greater in the SDD group than in the MDD group (Figure 2C, 2D). These results indicate that EGR1 expression is positively correlated with IVDD severity.

Previous studies have shown that oxidative stress is a pathological factor in IVDD progression, and TBHP is often used to construct oxidative stress-induced cell degeneration models *in vitro* [23–25]. Our study confirmed that compared with those in the control group, the intracellular ROS levels in the NPCs treated with TBHP were significantly greater, indicating that TBHP can induce oxidative stress in NPCs (Supplementary Figure 1). Therefore, we induced oxidative stress in NPCs using TBHP to construct an *in vitro* NPC degeneration model for subsequent experiments. In addition, we conducted Western blot experiments and showed that TBHP induced increased expression of EGR1 and the apoptosis-related protein Bax in NPCs and decreased expression of the antiapoptotic protein Bcl-2 and the ECM synthesis-related proteins aggrecan and collagen II, confirming that oxidative stress can lead to NPC apoptosis and the upregulation of EGR1 protein expression (Figure 2E–2J). Subsequently, qRT-PCR and immunofluorescence staining further verified that TBHP induced the upregulation of EGR1 mRNA and protein expression (Figure 2K, 2L). The above results indicate that TBHP induces an increase in ROS and apoptosis in NPCs and promotes the upregulation of EGR1 expression.

### EGR1 regulates apoptosis and ECM anabolism in NPCs

**Figure 3:**
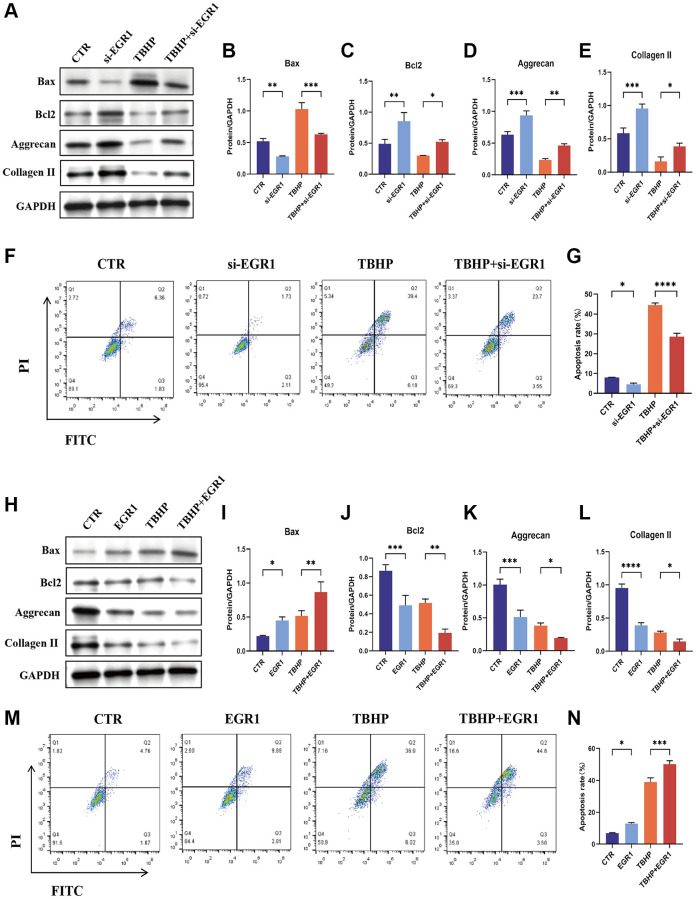
**EGR1 regulates apoptosis and ECM synthesis in NPCs.** (**A**) NPCs were transfected with 25 nM CTR siRNA or EGR1 siRNA-1 for 24 hours and then treated with 50 μM TBHP for 24 hours before protein extraction. The protein expression of Bax, Bcl-2, aggrecan, and collagen II was determined by western blotting. (**B**–**E**) Quantitative analysis of Bax, Bcl-2, aggrecan, and collagen II protein levels. (**F**) Representative flow cytometry images showing that EGR1 siRNA protected NPCs from TBHP-induced apoptosis. (**G**) Quantitative analysis of the NPC apoptosis rate. (**H**) NPCs were transfected with 5 μg of the EGR1 expression plasmid or the corresponding control vector. After 48 hours, the cells were treated with 50 μM TBHP for 24 hours. Total cellular proteins were extracted, and the expression of Bax, Bcl-2, aggrecan, and collagen II was determined by western blotting. (**I**–**L**) Quantitative analysis of Bax, Bcl-2, aggrecan, and collagen II protein levels. (**M**) Representative flow cytometry images showing that EGR1 overexpression increases TBHP-induced apoptosis in NPCs. (**N**) Quantitative analysis of the NPC apoptosis rate. The data are expressed as the mean ± SD (*n* = 3). ^*^p < 0.05, ^**^p < 0.01, ^***^p < 0.001, ^****^p < 0.0001.

We used loss-of-function and gain-of-function strategies to explore the role of EGR1 in NPCs. NPCs transfected with 3 sets of EGR1 siRNA and EGR1 siRNA1 (siEGR1-1) exhibited markedly reduced EGR1 mRNA expression (Supplementary Figure 2A). Therefore, EGR1-si1 was used in the subsequent experiments. We then examined the effect of EGR1 knockdown on TBHP-induced NPC apoptosis and impaired ECM synthesis. The results indicated that EGR1 knockdown significantly suppressed TBHP-induced Bax expression but significantly upregulated the protein expression of Bcl-2, aggrecan, and collagen II (Figure 3A–3E). The flow cytometry results showed that the percentage of apoptotic NPCs in the control group decreased, while that in the TBHP treatment group significantly increased, and EGR1 siRNA antagonized TBHP-induced NPC apoptosis (Figure 3F, 3G). Therefore, knocking down EGR1 can reduce TBHP-induced NPC apoptosis and increase ECM anabolism.

We also investigated the effects of EGR1 overexpression on TBHP-induced apoptosis and impaired ECM synthesis. As shown in Supplementary Figure 2B, the expression of EGR1 was increased considerably in the overexpression group compared with that in the Control (CTR) group. Western blotting results indicated that EGR1 overexpression significantly increased TBHP-induced Bax expression but significantly decreased the protein levels of Bcl-2, aggrecan, and collagen II (Figure 3H–3L). Moreover, the flow cytometry results demonstrated that EGR1 overexpression increased TBHP-induced NPC apoptosis (Figure 3M, 3N). Taken together, these findings indicate that EGR1 overexpression can promote TBHP-induced NPC apoptosis and reduce ECM anabolic metabolism.

### NR4A3 is a downstream target gene of EGR1

**Figure 4:**
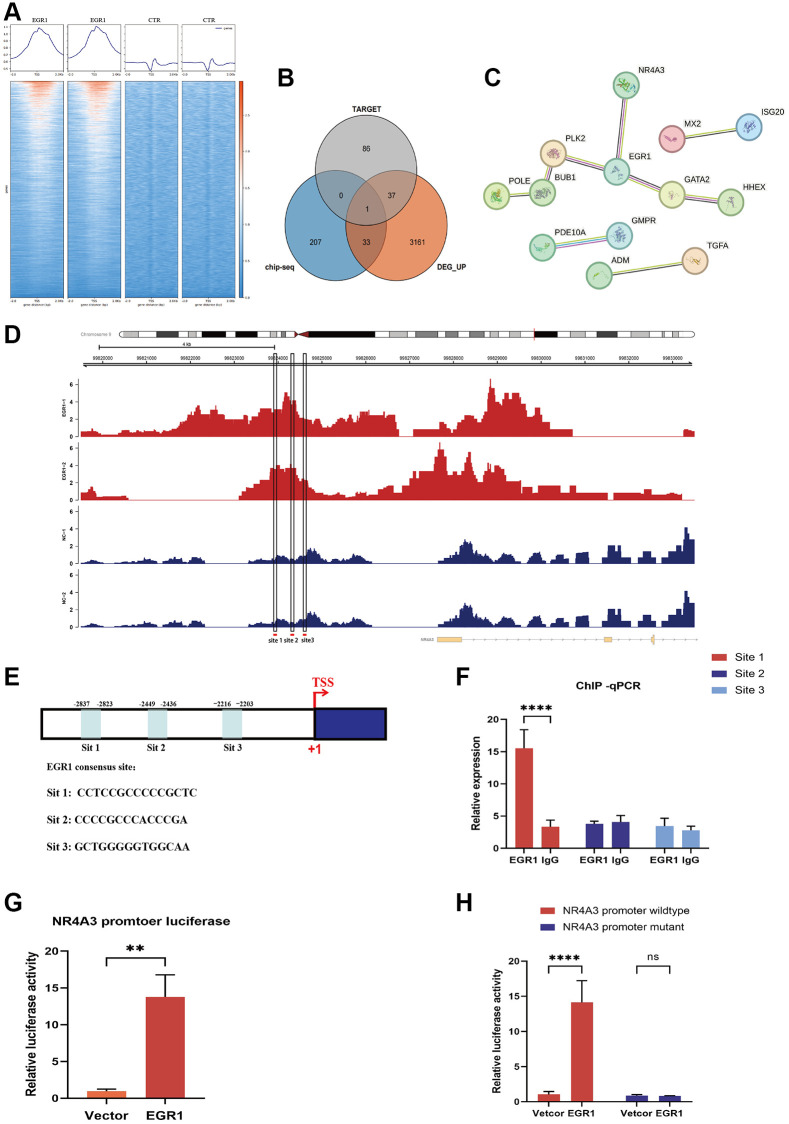
**RNA-seq and ChIP-seq were performed to screen the downstream target genes of EGR1.** (**A**) Heatmap analysis was performed using DeepTools software to compare the signal intensity adjacent to the transcription start site between the EGR1 overexpression and control groups. (**B**) Venn diagram showing screened candidate genes identified by the intersection of Chip-seq and RNA-seq data. (**C**) Protein-protein interaction analysis indicating interactions between candidate genes and EGR1. (**D**) Potential binding sites for EGR1 on the nuclear receptor 4A3 (NR4A3) promoter were identified using the JASPAR database to analyze the ChIP-seq data. (**E**) Schematic diagram of three potential EGR1 binding sites (site 1, site 2, and site 3) in the NR4A3 promoter. (**F**) ChIP-qPCR was utilized to assess the binding of EGR1 to the NR4A3 promoter region. (**G**) The activation effect of EGR1 on the NR4A3 promoter was evaluated using a dual-luciferase reporter assay. (**H**) NPCs were transfected with plasmids containing either the full-length or site 1-mutated NR4A3 promoter to measure the luciferase activity of the NR4A3 promoter upon EGR1 overexpression. The data are expressed as the mean ± SD (*n* = 3). ^**^p < 0.01, ^****^p < 0.0001.

To explore the downstream target genes of EGR1, we screened target genes of EGR1 in human NPCs using ChIP-seq combined with RNA-seq technology. We performed a heatmap analysis of the ChIP-seq results using DeepTools software and found that the signal intensity near the transcription start site was significantly greater in the EGR1 high-expression group than that in the control group, indicating that EGR1 can bind to the promoter sequences of its target genes to exert its function in NPCs (Figure 4A). Next, we identified 32 candidate genes by intersecting 241 potential binding genes from the ChIP-seq data with 3,232 differentially upregulated genes from the RNA-seq data, excluding previously reported EGR1 target genes (Figure 4B). We constructed a protein-protein interaction network for these 32 genes and found that NR4A3, GATA2, and PLK2 strongly interact with EGR1 (Figure 4C). According to existing research reports, NR4A3 is involved in various apoptotic signaling pathways in cells [18, 26, 27]. Therefore, we speculate that NR4A3 may be a target gene through which EGR1 regulates NPC apoptosis.

To validate our hypothesis, we conducted a detailed analysis of the ChIP-seq data and identified significant peak regions near the NR4A3 promoter sequence (Figure 4D). Furthermore, by utilizing the transcription factor-binding site database JASPAR (https://jaspar.genereg.net/), we selected the top three binding site sequences for subsequent experimental validation according to their scores. Three potential binding sites were identified: site 1, CCTCCGCCCCCGCTC; site 2, CCCCGCCCACCCGA; and site 3, GCTGGGGGTGGCAA (Figure 4E). To determine which NR4A3 promoter site is specifically modulated by EGR1, we performed ChIP-qPCR experiments and identified site 1 as the critical locus for EGR1-mediated transcriptional regulation of NR4A3, while sites 2 and 3 did not regulate NR4A3 expression (Figure 4F). To further substantiate this conclusion, we constructed luciferase reporter vectors containing full-length and site 1-mutated NR4A3 promoter sequences and utilized a dual-luciferase reporter assay system to assess the impact of EGR1 overexpression on luciferase activity. The results demonstrated that EGR1 overexpression significantly enhanced the luciferase activity of the NR4A3 promoter region (Figure 4G). However, when site 1 within the NR4A3 promoter was mutated, EGR1 no longer affected promoter activity (Figure 4H). Collectively, these findings indicate that NR4A3 is a direct transcriptional target of EGR1, with EGR1 modulating NR4A3 transcriptional activity by binding to the NR4A3 promoter region.

**Figure 5:**
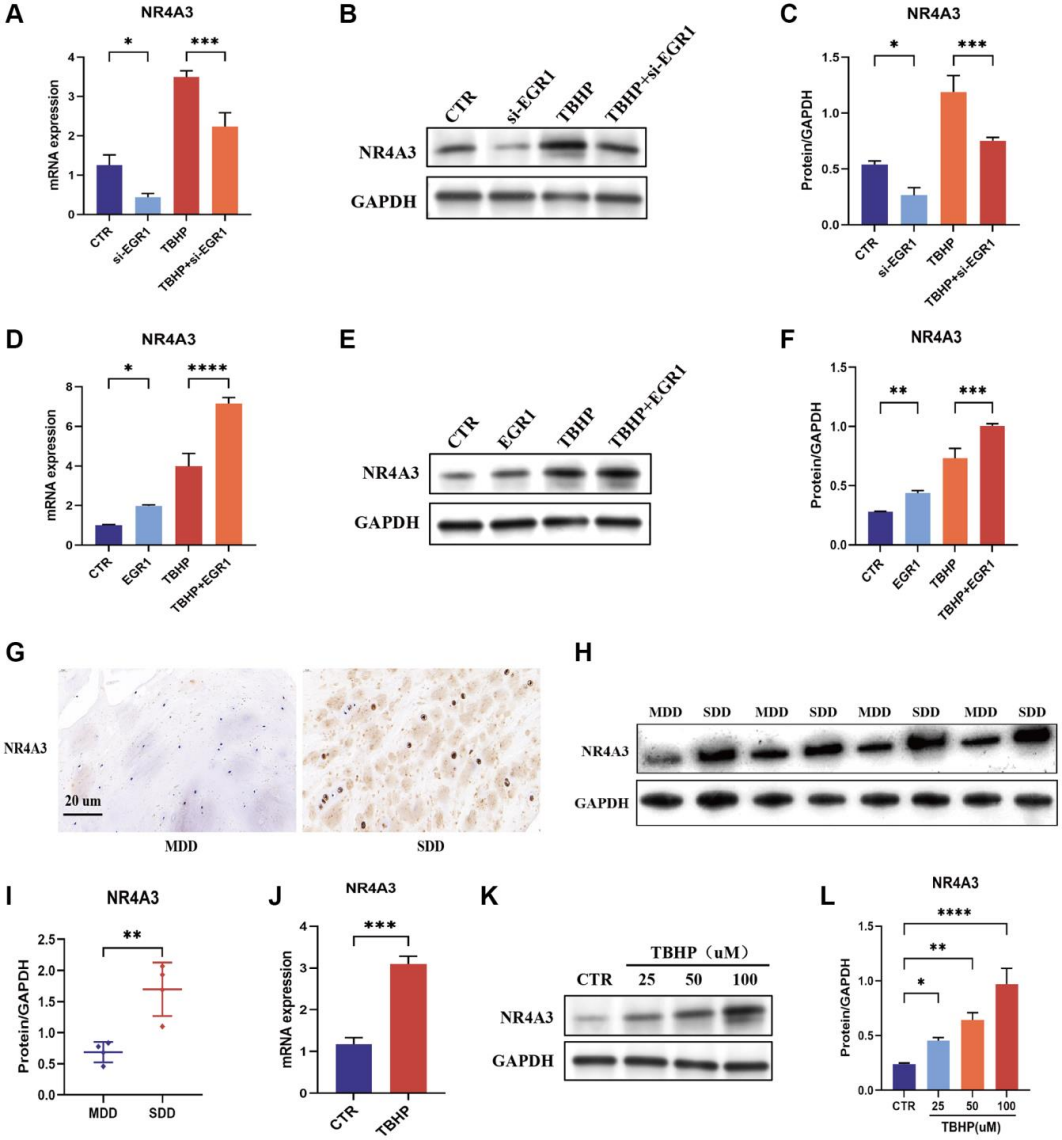
**EGR1 positively regulates the expression of NR4A3.** (**A**–**C**) NPCs were transfected with 25 nM Control siRNA or EGR1 siRNA-1 for 24 hours and then treated with 50 μM TBHP for 24 hours before protein or mRNA extraction. The mRNA and protein expression levels of NR4A3 were determined by qRT-PCR and Western blotting. (**D**–**F**) NPCs were transfected with 5 μg of the EGR1 expression plasmid or its control vector for 24 hours and then treated with 50 μM TBHP for 24 hours before protein or mRNA extraction. The mRNA and protein expression of NR4A3 was determined by qRT-PCR and Western blotting. (**G**) The expression of NR4A3 in SDD and MDD patients was examined by IHC staining. (**H**) The protein expression of NR4A3 in SDD and MDD was examined by Western blotting. (**I**) Quantitative analysis of NR4A3 protein level in SDD and MDD. (**J**) The mRNA expression of NR4A3 in TBHP-treated NPCs was examined by qRT-PCR. (**K**) NPCs were treated with 0, 25, 50, or 100 μM TBHP for 24 h, after which the protein was extracted, and the NR4A3 expression level was determined by Western blotting. (**L**) Quantitative analysis of the NR4A3 protein level. ^*^p < 0.05, ^**^p < 0.01, ^***^p < 0.001, ^****^p < 0.0001.

To further explore EGR1 regulation of NR4A3, we next examined the effect of EGR1 knockdown or overexpression on NR4A3 expression. The results indicated that EGR1 knockdown significantly inhibited NR4A3 mRNA and protein expression and TBHP-induced NR4A3 mRNA and protein expression (Figure 5A–5C). In contrast, EGR1 overexpression markedly increased NR4A3 mRNA and protein expression and TBHP-induced NR4A3 mRNA and protein expression (Figure 5D–5F). Taken together, these results suggest that EGR1 positively regulates the expression of NR4A3.

### NR4A3 expression is upregulated in human degenerative intervertebral discs and TBHP-treated NPCs

IHC staining revealed that the expression of NR4A3 was significantly greater in the SDD group than in the MDD group (Figure 5G). These findings were further confirmed by western blotting (Figure 5H, 5I). These results indicate that NR4A3 expression is positively correlated with IVDD severity. Moreover, qRT-PCR showed that TBHP induced NR4A3 mRNA expression in NPCs (Figure 5J). Similarly, TBHP induced NR4A3 protein expression in a concentration-dependent manner in NPCs (Figure 5K, 5L). Overall, these findings demonstrate that NR4A3 expression is upregulated in human degenerative intervertebral discs and TBHP-induced NPCs.

### NR4A3 regulates apoptosis and ECM anabolism in NPCs

**Figure 6:**
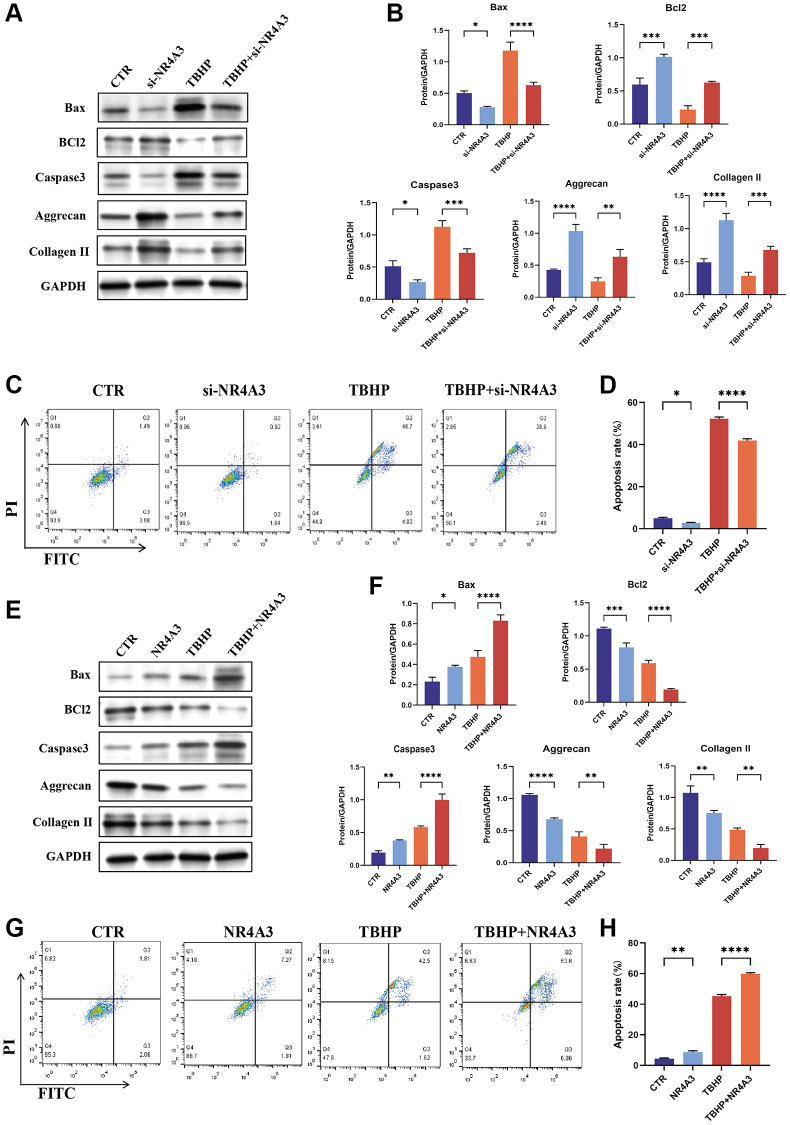
**NR4A3 regulates NPC apoptosis and ECM synthesis.** (**A**) NPCs were transfected with 25 nM CTR siRNA or NR4A3 siRNA-1 for 24 hours and then treated with 50 μM TBHP for 24 hours before protein extraction. The protein expression levels of Bax, Bcl-2, caspase 3, aggrecan, and collagen II were determined by western blotting. (**B**) Quantitative analysis of Bax, Bcl-2, caspase 3, aggrecan, and collagen II protein levels. (**C**) Representative flow cytometry images showing that NR4A3 siRNA protected NPCs from TBHP-induced apoptosis. (**D**) Quantitative analysis of the NPC apoptosis rate. (**E**) NPCs were transfected with 5 μg of the NR4A3 expression plasmid or the corresponding control vector. After 48 hours, the cells were treated with 50 μM TBHP for 24 hours. Total cellular proteins were extracted, and the expression levels of Bax, Bcl-2, caspase-3, aggrecan, and collagen II were determined by western blotting. (**F**) Quantitative analysis of Bax, Bcl-2, caspase-3, aggrecan, and collagen II protein levels. (**G**) Representative flow cytometry images showing that overexpression of NR4A3 increased TBHP-induced apoptosis in NPCs. (**H**) Quantitative analysis of the NPC apoptosis rate. The data are expressed as the mean ± SD (*n* = 3). ^*^p < 0.05, ^**^p < 0.01, ^***^p < 0.001, ^****^p < 0.0001.

To investigate the function of NR4A3 in NPCs, NR4A3 was knocked down and overexpressed. NR4A3-si1 knocked down NR4A3 more effectively than the other siRNAs (Supplementary Figure 3A). Therefore, NR4A3-si1 was used in subsequent knockdown experiments. We evaluated the impact of NR4A3 knockdown on TBHP-induced cell apoptosis and impaired ECM synthesis. The results indicated that NR4A3 knockdown strongly downregulated TBHP-induced Bax and Caspase 3 but markedly upregulated Bcl-2, aggrecan, and collagen II protein levels (Figure 6A, 6B). Subsequent flow cytometry analysis showed that apoptosis was significantly increased in the TBHP-treated group, while NR4A3 knockdown partly prevented TBHP-induced apoptosis in NPCs (Figure 6C, 6D).

To further confirm these results, NPCs were transiently transfected with an NR4A3 overexpression plasmid (Supplementary Figure 3B). The results indicated that the overexpression of NR4A3 strongly increased the TBHP-induced increase in Bax and Caspase 3 but decreased the protein levels of Bcl-2, aggrecan, and collagen II (Figure 6E, 6F). Moreover, the flow cytometry results demonstrated that NR4A3 overexpression increased TBHP-induced NPC apoptosis (Figure 6G, 6H). Taken together, these findings indicate that NR4A3 can regulate apoptosis and ECM anabolism in NPCs.

### EGR1 promotes NPC apoptosis in an NR4A3-dependent manner

**Figure 7:**
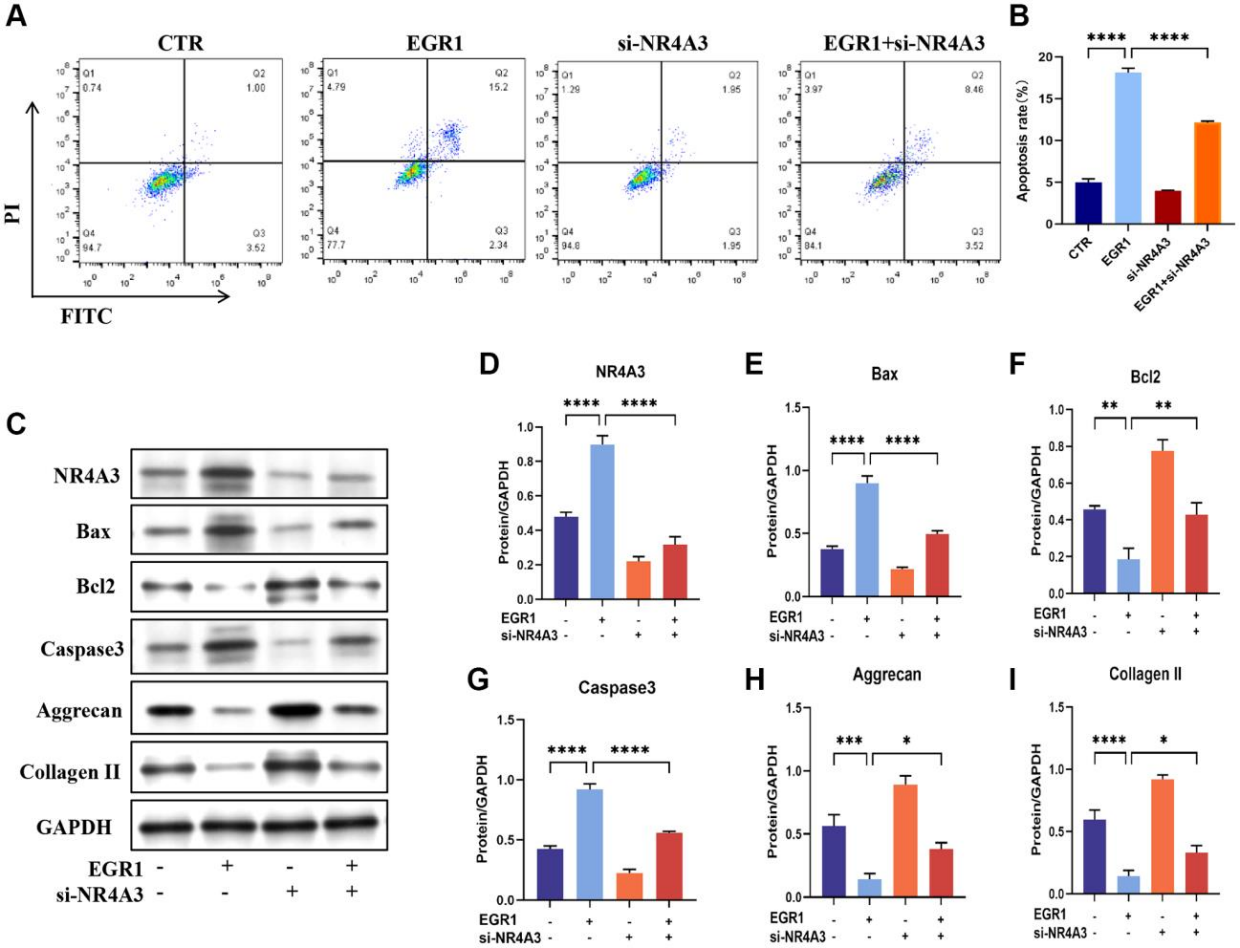
**NR4A3 is necessary for EGR1-induced apoptosis and the impairment of ECM synthesis metabolism in human NPCs *in vitro*.** (**A**) NPCs were transfected with EGR1 overexpression plasmids and NR4A3 siRNA, followed by flow cytometry analysis to determine the percentage of apoptotic cells in the different groups. (**B**) Quantitative analysis of the NPC apoptosis rate. (**C**) Western blot analysis was performed to determine the protein expression levels of NR4A3, Bax, Bcl-2, caspase-3, aggrecan, and collagen II in the different treatment groups. (**D**–**I**) Quantitative analysis of the NR4A3, Bax, Bcl-2, caspase-3, aggrecan, and collagen II protein levels. The data are expressed as the mean ± SD (*n* = 3). ^*^p < 0.05, ^**^p < 0.01, ^***^p < 0.001, ^****^p < 0.0001.

Given the above results, we speculated that NR4A3 functions as an effector of EGR1 in IVDD progression. We then knocked down NR4A3 in NPCs overexpressing EGR1. Flow cytometry demonstrated that knocking down NR4A3 significantly alleviated the EGR1 overexpression-induced NPC apoptosis. (Figure 7A, 7B). To confirm this conclusion, we further examined the effect of NR4A3 knockdown on EGR1 overexpression-induced apoptosis and impaired ECM anabolism by western blotting. NR4A3 knockdown strongly downregulated EGR1 overexpression-induced Bax and Caspase 3 but markedly upregulated EGR1 overexpression-inhibited Bcl-2, aggrecan, and collagen II protein levels (Figure 7C–7I). In summary, these results suggest that the promotion of apoptosis and impaired ECM synthesis metabolism in NPCs by EGR1 is contingent upon NR4A3 expression.

### EGR1 siRNA alleviates the progression of IVDD by regulating NR4A3 in an IVDD rat model

**Figure 8:**
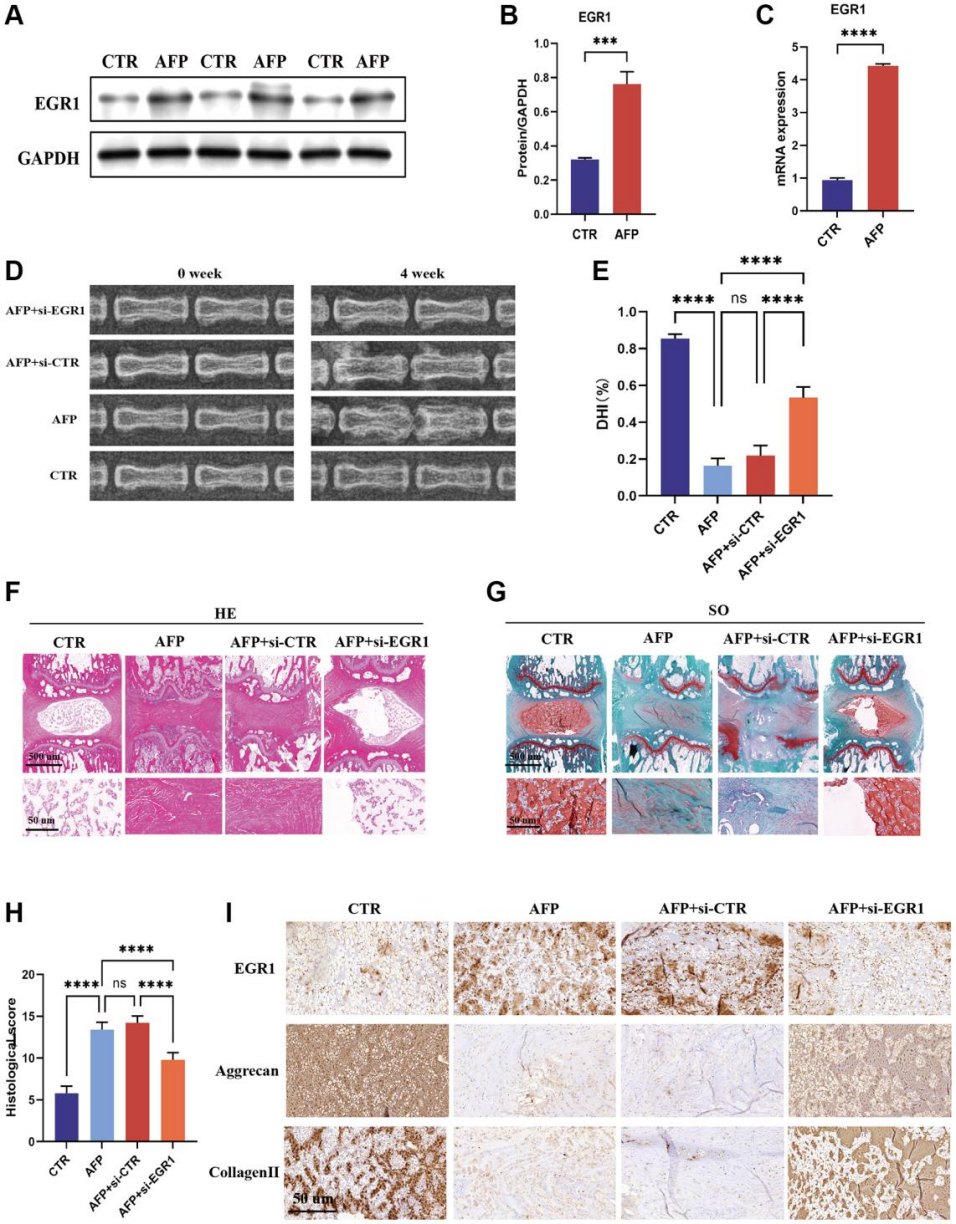
**EGR1 siRNA alleviates the progression of IVDD in the AFP-treated rat model.** (**A**) The protein expression of EGR1 in the IVDD rat model was determined by western blotting. (**B**) Quantitative analysis of EGR1 levels. (**C**) EGR1 mRNA expression in the IVDD rat model was examined by qRT-PCR. (**D**) X-ray images of the different groups before and 4 weeks after puncture. (**E**) Changes in intervertebral disc height were evaluated. (**F**, **G**) Representative images of HE and safranin-O staining in the four groups. (**H**) Histological scores were calculated. (**I**) Immunohistochemical staining of EGR1, aggrecan, and collagen II. ^***^p < 0.001, ^****^p < 0.0001.

**Figure 9:**
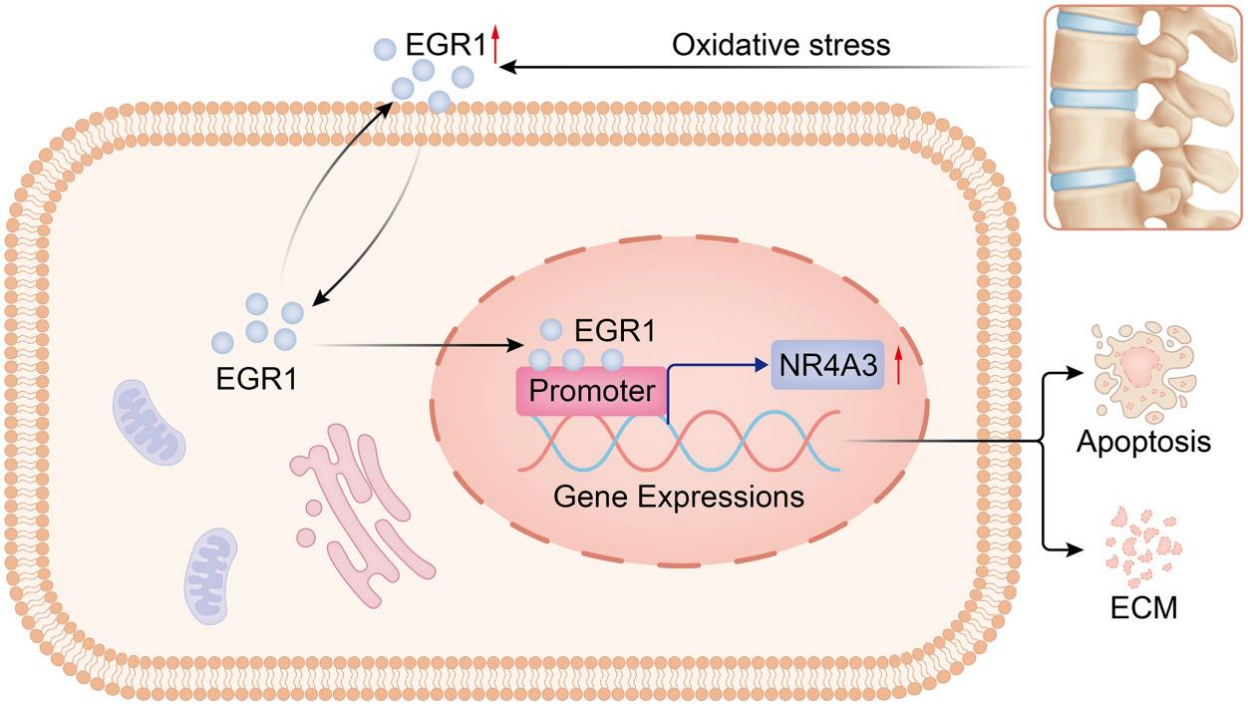
**Diagram of the molecular mechanism by which EGR1 regulates NR4A3 in IVDD.** By regulating NR4A3, EGR1 accelerates NPC apoptosis and impairs extracellular matrix anabolism, thereby promoting the progression of IVDD.

To determine the role of EGR1 in the progression of IVDD *in vivo*, we injected EGR1 siRNA into an IVDD rat model induced by annulus fibrosus puncture (AFP). First, we examined the changes in EGR1 expression in IVDD. EGR1 protein and mRNA expression was greater in the AFP group than in the control group (Figure 8A–8C). Furthermore, an X-ray was performed 4 weeks after surgery (Figure 8D), and the results showed that the percentage of DHI in the AFP+si-EGR1 group was significantly greater than that in the AFP and AFP+si-CTR groups (Figure 8E). This result suggested that EGR1 siRNA could slow the progression of IVDD *in vivo*. Consistent with the imaging results, H&E and S-O staining revealed a relatively indistinct boundary between the NP and the AF and a decreased NPC number in the AFP and AFP+si-CTR groups, and this effect was alleviated in IVDD model rats treated with EGR1 siRNA (Figure 8F–8H). Similarly, IHC analysis indicated that the expression levels of aggrecan and collagen II in the AFP+si-EGR1 group were elevated relative to those in the AFP + si-CTR group (Figure 8I). Overall, these results suggest that inhibition of EGR1 has the potential to alleviate the progression of IVDD (Figure 9).

## DISCUSSION

LBP is a globally recognized issue of significant academic interest and a pressing challenge in global public health. IVDD is the main cause of LBP [28]. The treatment of IVDD includes conservative and surgical strategies, but it is difficult to successfully resolve the underlying biological problem [29]. Therefore, there is an urgent need for new treatments to combat IVDD. We explored potential therapeutic targets of IVDD using bioinformatics analyses, revealing abnormal expression of EGR1 in this disease. Previous studies have shown that EGR1 is upregulated in IVDD, but the underlying mechanism is unclear [30]. Our study confirmed that NR4A3 is a direct target of EGR1 and that EGR1 regulates its transcription. Moreover, the role of EGR1 in regulating NPC apoptosis and ECM synthesis depends on NR4A3. Finally, we identified the inhibitory effect of EGR1 siRNA on the progression of IVDD *in vivo*.

The early growth response protein family is categorized by an identical protein organization encompassing four family numbers: EGR-1, EGR-2, EGR-3, and EGR-4 [31, 32]. An identical protein organization is characterized by three conserved zinc finger regions in the C-terminus that interact with target genes that harbor specific GC-rich consensus sequences [33]. The transcriptional activation domain, oriented at the N-terminus, contains binding sites for other proteins that augment the transcriptional control of EGR1. Many studies have shown that EGR1 is abnormally expressed in various diseases, such as tumors, central nervous system diseases, and cardiovascular and inflammatory diseases [34–36]. We analyzed NP samples from patients by WB and IHC and demonstrated that EGR1 expression levels positively correlate with IVDD severity. Notably, EGR1 is involved in various key biological processes as a transcription factor, such as apoptosis, proliferation, ECM metabolism, and oxidative stress [37]. For example, in prostate cells lacking the TP53 gene, EGR1 is believed to promote apoptosis by activating TNF-α [38]. During keloid formation, EGR1 responds to TGF-β and promotes ROS production by activating NADPH oxidase 4 (NOX4) [39]. Furthermore, EGR1 overexpression triggers ECM catabolism in specific embryonic fibroblasts in mice [40]. Our study also confirmed that EGR1 is significantly increased in oxidative stress-induced degenerative NPCs. Moreover, KEGG pathway analysis revealed that EGR1 upregulation is associated with the apoptosis pathway. After knocking down EGR1, the apoptosis rate in degenerative NPCs was significantly lower than that in control NPCs, and EGR1 overexpression had the opposite effect. Notably, EGR1 siRNA effectively relieved IVDD in the surgery induced IVDD rat model. Overall, these findings suggest that EGR1 is a potential therapeutic target for IVDD. Although the therapeutic effect of EGR1 siRNA on a surgery induced IVDD rat model is beneficial, the optimal dose remains to be determined, and the side effects are still unknown. Therefore, we will investigate the optimal dose and side effects in future work.

The transcription factor EGR1 has many target genes, including SOX9, SNAI2, CCND1, ABCA2, and ATF3, some of which are involved in apoptosis [41–43]. To elucidate the modulatory mechanism of EGR1 in IVDD progression, we screened the potential EGR1 target gene NR4A3 by ChIP-seq combined with RNA-seq. We found a strong interaction between NR4A3 and EGR1, which was confirmed by ChIP-qPCR and luciferase assays. NR4A3 is an immediate early gene induced by diverse stimuli, including peptide hormones, growth modulators, inflammatory cues, physiological triggers, and cellular distress [44]. The cellular levels of NR4A3 are tightly controlled under normal physiological conditions, and NR4A3 is transiently induced and activated by a diverse variety of extracellular signals, including genotoxic cellular stress, hormones, inflammatory cytokines, and metabolic, mitogenic, and apoptotic signals [45–47]. *NR4A3* plays a central role in the negative selection of T lymphocytes and surface IgM-mediated and viral-induced B-cell apoptosis [48, 49]. *NR4A*3 has also been implicated in the regulation of proliferation, apoptosis, and cell cycle arrest in cancer cells and plays a major role in the apoptotic responses of epithelial cancer cells, including their sensitivity to antineoplastic agents [50, 51]. In the present study, we demonstrated that NR4A3 is a downstream target of EGR1 and is directly regulated by EGR1 through interactions with binding site 1 in the NR4A3 promoter. In addition, EGR1 promoted NPC apoptosis in an NR4A3-dependent manner; NR4A3 silencing reversed the effect of EGR1 on NP cell apoptosis. However, *in vivo* experiments are needed to clarify the effect of NR4A3 on IVDD. Overall, our results provide new insight into how EGR1 promotes apoptosis by regulating NR4A3.

## CONCLUSION

In summary, our results showed that EGR1 knockdown could decrease the expression of NR4A3, reducing NPC apoptosis and maintaining the stability of the ECM, thereby inhibiting IVDD progression. Our research links EGR1 with NR4A3 for the first time and demonstrates the molecular mechanism regulating IVDD. In brief, our results reveal that the EGR1-NR4A3 axis may be a potential therapeutic target in IVDD therapy.

## Supplementary Materials

Supplementary Figures

Supplementary Tables
