# Supplementary material for: Oxidative stress-induced EGR1 upregulation promotes NR4A3-mediated nucleus pulposus cells apoptosis in intervertebral disc degeneration
Source: Aging (Albany NY). 2024 Jun 28;16(12):10216–38. doi: 10.18632/aging.205920 (PMC11236312; doi:10.18632/aging.205920)
Supplement: Supplementary Figures [file aging-16-205920-s001.pdf]

SUPPLEMENTARY FIGURES

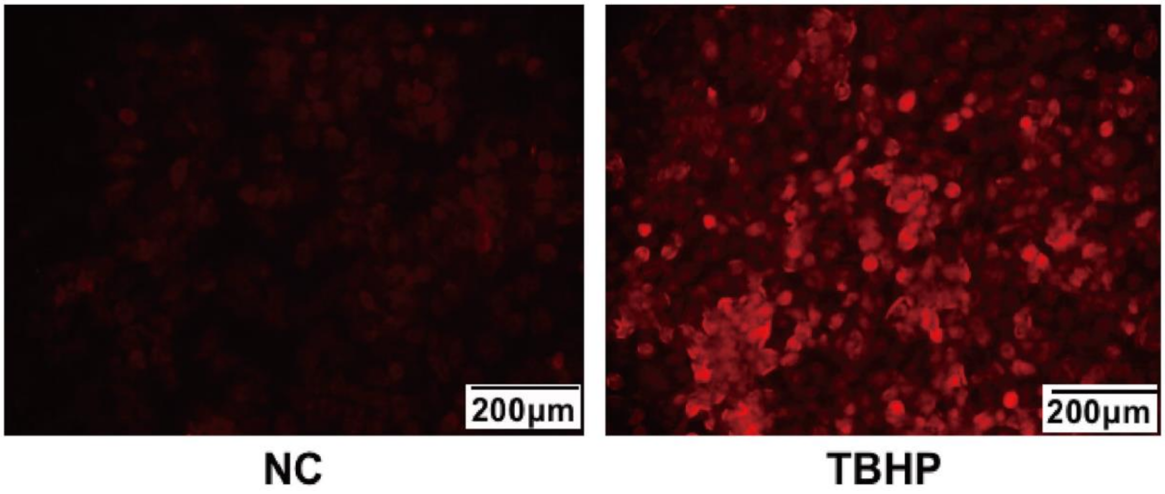

Supplementary Figure 1. Reactive oxygen species levels in NPCs. The control group consisted of normal NPCs, while the experimental group consisted of NPCs induced with TBHP (50 μM, 24 h). The difference in reactive oxygen species levels between the two groups was compared under a fluorescence microscope.

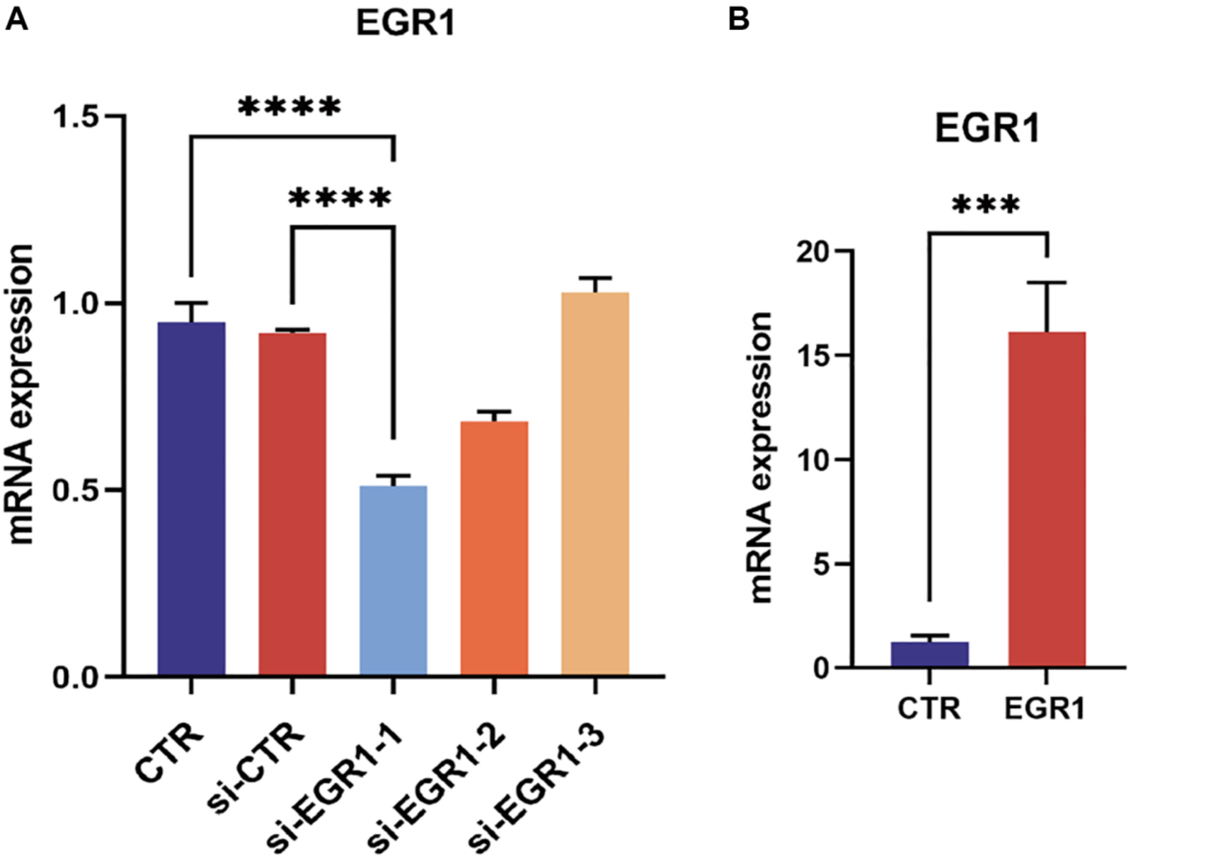

Supplementary Figure 2. Efficiency of EGR1 knockdown and overexpression. (**A**) mRNA levels in NPCs after transfection with different EGR1 siRNAs. (**B**) mRNA levels in NPCs after transient transfection with plasmids overexpressing EGR1. The data are expressed as the mean ± SD (n = 3). $^{***}p < 0.001$, $^{****}p < 0.0001$.

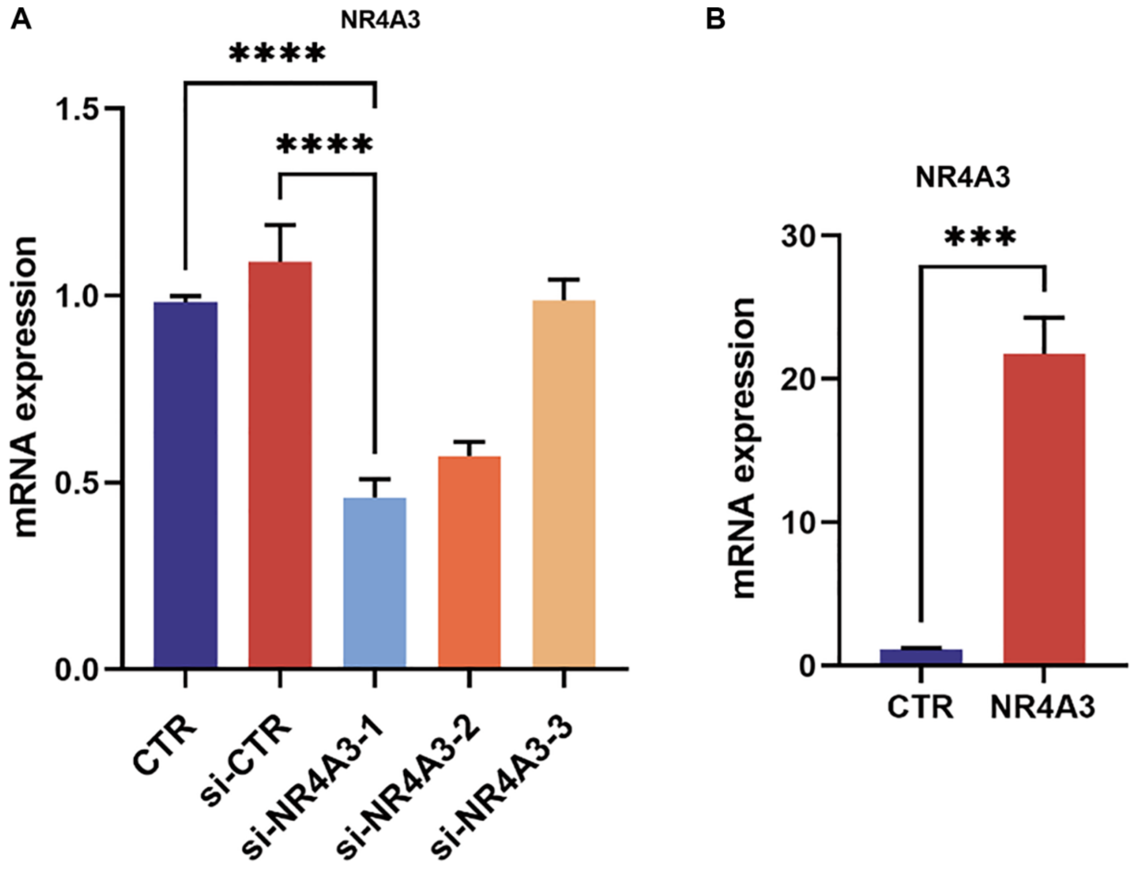

Supplementary Figure 3. Efficiency of NR4A3 knockdown and overexpression. (**A**) mRNA levels in NPCs after transfection with different NR4A3 siRNAs. (**B**) mRNA levels in NPCs after transient transfection with plasmids overexpressing NR4A3. The data are expressed as the mean ± SD (n = 3). $^{***}p < 0.001$, $^{****}p < 0.0001$.
