# Supplementary material for: Oxidative stress-induced EGR1 upregulation promotes NR4A3-mediated nucleus pulposus cells apoptosis in intervertebral disc degeneration
Source: Aging (Albany NY). 2024 Jun 28;16(12):10216–38. doi: 10.18632/aging.205920 (PMC11236312; doi:10.18632/aging.205920)
Supplement: Supplementary Tables [file aging-16-205920-s002.pdf]

SUPPLEMENTARY TABLES

Supplementary Table 1. Information on human disc samples from 10 patients.

Samples	Diagnosis	Sex	Age	BMI	Level	Grade
1	Acute disc herniation	M	19y	23.7	L4/L5	II
2	Acute disc herniation	M	18y	24.1	L4/L5	I
3	Acute disc herniation	M	28y	25.3	L4/L5	II
4	Spinal fractures	F	33y	20.7	T12/L1	I
5	Acute disc herniation	F	16y	23.5	L5/S1	I
6	Degenerative disc disease	F	51y	27.5	L5/S1	III
7	Degenerative disc disease	F	80y	22.1	L4/L5	V
8	Degenerative disc disease	M	66y	26.4	L4/L5	V
9	Degenerative disc disease	M	67y	18.4	L4/L5	V
10	Degenerative disc disease	M	62y	26.2	L5/S1	IV

Abbreviations: M: male; F: female, y: year.

Supplementary Table 2. Primer sequence for ChIP-qPCR.

Site	Gene	Primer sequence
Site 1	NR4A3-ChIP-F1	GAATCTCAGTCTTTCCCTTTCTGTC
	NR4A3-ChIP-R1	TGGGCAGACGAAAGGAACAC
Site 2	NR4A3-ChIP-F2	GAGGAAAGGCTGTGTGGGTC
	NR4A3-ChIP-R2	GCATTCCCTGAGGGCCTTTC
Site 3	NR4A3-ChIP-F3	TGGGAAGATCCGCTTCTACC
	NR4A3-ChIP-R3	GAGAAAGGGACTCGGGGCTC
